# High stakes, low information, no roads: a qualitative evaluation of determinants of air ambulance utilization and decision making in rural Alaska

**DOI:** 10.64898/2026.09.21.26363602

**Published:** 2026-09-23

**Authors:** Brian Rice, Brandon A. Berger, Robert Onders, Sar Medoff, Henry Saul

## Abstract

**Background:**

In rural Alaska, where most communities lack road access, air ambulances serve as a critical link between remote communities and hospital services.

**Objective:**

To examine how air ambulance utilization decisions are made within the rural Alaskan health organization ecosystem.

**Methods:**

We conducted semi-structured interviews with five key stakeholders. Transcripts were analyzed thematically using the Practical, Robust Implementation and Sustainability Model (PRISM).

**Results:**

Four themes emerged that collectively define the structure of the air ambulance decision-making system, each reflecting interactions across multiple PRISM domains: limited formal guidance, logistical constraints affecting transport options, variability driven by communication and clinician risk tolerance, and reliance on unwritten rules. Together, these describe decision-making in a limited information state with incomplete feedback, leading to experiential and locally evolved practices.

**Conclusion:**

Air ambulance use reflects clinical–logistical constraints rather than critical illness alone. Linking transport decisions to outcomes and developing structured decision support may improve consistency and optimize care delivery.

## Full Text Availability

The license terms selected by the author(s) for this preprint version do not permit archiving in PMC. The full text is available from the preprint server.

